# Crystal structure of 1,2-bis­((benzyl­sulfan­yl){2-[1-(2-hy­droxy­phen­yl)ethyl­idene]hydrazin-1-yl­idene}meth­yl)disulfane

**DOI:** 10.1107/S2056989016002371

**Published:** 2016-02-17

**Authors:** M. A. A. A. A. Islam, M. C. Sheikh, M. H. Islam, R. Miyatake, E. Zangrando

**Affiliations:** a Department of Chemistry, Rajshahi University of Engineering & Technology, Rajshahi 6204, Bangladesh; b Department of Applied Chemistry, Faculty of Engineering, University of Toyama, 3190 Gofuku, Toyama 930-8555, Japan; c Center for Environmental Conservation and Research Safety, University of Toyama, 3190 Gofuku, Toyama 930-8555, Japan; d Department of Chemical and Pharmaceutical Sciences, via Giorgieri 1, 34127 Trieste, Italy

**Keywords:** crystal structure, Schiff base, di­thio­carbazate, hy­droxy­phenyl­ethyl­idene, hydrogen bonds

## Abstract

The title compound, C_32_H_30_N_4_O_2_S_4_, consists of two Schiff base moieties, namely two *S*-benzyl-β-*N*-(2-hy­droxy­phenyl­ethyl­idene)di­thio­carbazate groups, which are connected through an S—S single bond. These two moieties are twisted with respect to each other, with a dihedral angle of 87.88 (4)° between the S_2_C=N planes. A bifurcated intra­molecular O—H⋯(N,S) hydrogen bond is observed in each moiety. In the crystal, mol­ecules are linked by pairs of C—H⋯O hydrogen bonds into inversion dimers. The dimers are further stacked in a column along the *b* axis through weak C—H⋯π inter­actions.

## Abstract 2

The title mol­ecule consists of two Schiff base moieties, namely two *S*-benzyl-β-*N*-(2-hy­droxy­phenyl­ethyl­idene)di­thio­carbazate groups, connected through an S—S single bond. The two moieties are twisted with a dihedral angle of 87.88 (4)° between the S_2_C=N planes.

## Chemical context   

There has been immense inter­est in nitro­gen–sulfur donor ligands since the report on *S*-benzyl­dithio­carbazate (SBDTC) (Ali & Tarafder, 1977 ▸). Since then, a number of Schiff bases have been derived from SBDTC (Crouse *et al.*, 2004 ▸; Howlader *et al.*, 2015 ▸). The versatile coordination chemistry and increasingly important biological properties of ligands derived from SBDTC have also received much attention (Zangrando *et al.*, 2015 ▸). In a continuation of our research in this area, the title compound (systematic name: 2-[1-(2-{(benzyl­sulfan­yl)[((benzyl­sulfan­yl){2-[1-(2-hy­droxy­phen­yl)ethyl­idene]hydrazin-1-yl­idene}meth­yl)disulfan­yl]methylidene}eth­yl]phenol) was prepared from SBDTC.
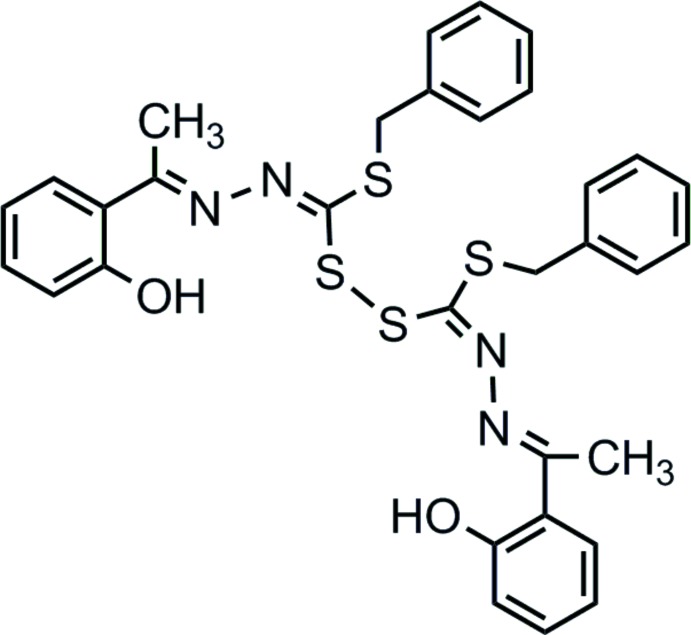


## Structural commentary   

**Figure 1:**
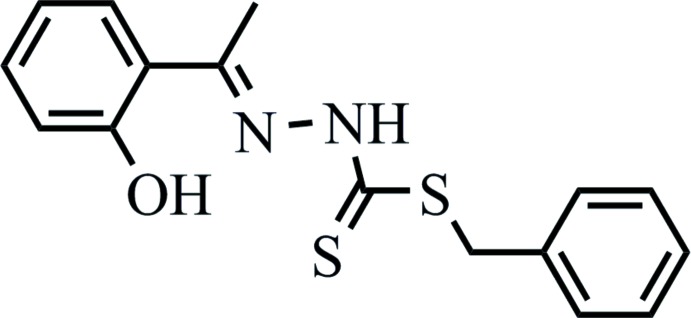
Chemical scheme of *S*-benzyl-β-*N*-(2-hy­droxy­phenyl­ethyl­idene)di­thio­carbazate (systematic name: benzyl 2-[1-(2-hy­droxy­phen­yl)ethyl­idene]hydrazinecarbodi­thio­ate).

**Figure 2:**
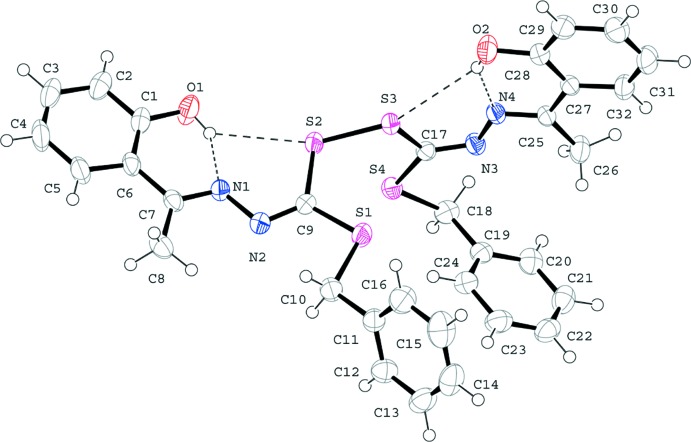
The mol­ecular structure of the title compound, showing 50% probability displacement ellipsoids and the atom numbering. H atoms are drawn as circles of arbitrary size. O—H⋯N and O—H⋯S hydrogen bonds are indicated by dashed lines.

In the title compound, the arrangement of the two Schiff base moieties (Fig. 1 ▸) is almost orthogonal with respect to the S2—S3 thio­ether bond (Fig. 2 ▸). The S2—S3 bond distance of 2.0373 (4) Å lies just within the range of S—S single-bond lengths (2.03–2.36 Å) (Knop *et al.*, 1988 ▸). In each of the Schiff base moieties, the benzene ring and the di­thio­carbazate fragment are arranged *trans* across the C=N bond (C7=N1 and C25=N4). The (imino­eth­yl)phenol fragments (C1–C8/O1/N1 and C25–C32/O2/N4) are essentially planar with maximum deviations of 0.0559 (12) Å for N1 and 0.0200 (11) Å for N4 and make dihedral angles of 18.17 (4) and 17.49 (4)° with the N2/S1/S2/C9 and N3/S3/S4/C17 planes, respectively. The C—S distances (C9—S1, C9—S2, C10—S1, C17—S4, C17—S3 and C18—S4) of 1.7461 (12)–1.8220 (13) Å are comparable to the values for the most similar di­thio­carbazate derivatives (Zangrando *et al.*, 2015 ▸; Crouse *et al.*, 2004 ▸). The C—N distances (C7—N1, C9—N2, C25—N4 and C17—N3) of 1.2789 (15)–1.2983 (15) Å indicate double-bond character (Tarafder *et al.*, 2008 ▸), but they are slightly shorter than the C=N bond of the *S*-2-picolyl di­thio­carbazate Schiff base of 2-acetyl pyrrole (Crouse *et al.*, 2004 ▸). The bond angles S1—C9—S2 [117.77 (6)°], S2—C9—N2 [120.78 (9)°], S3—C17—S4 [118.82 (7)°] and S3—C17—N3 [120.15 (12)°] are also comparable with those observed in *trans*-*cis S*-benzyl di­thio­carbazate (Tarafder *et al.*, 2008 ▸). Intra­molecular O—H⋯N and O—H⋯S hydrogen bonds are observed (Table 1 ▸).

## Supra­molecular features   

**Figure 3:**
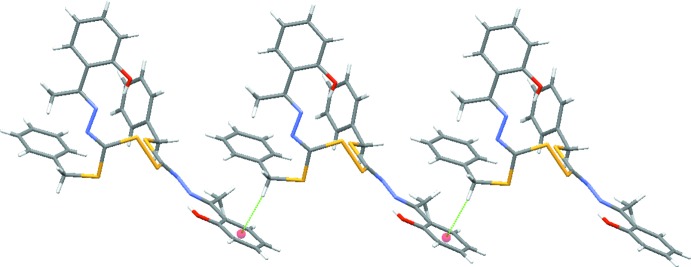
A packing diagram of the title compound. The C—H⋯π inter­actions are shown as green lines.

Pairs of inter­molecular C—H⋯O hydrogen bonds (Table 1 ▸) link the mol­ecules into inversion dimers. C—H⋯π inter­actions are also observed in the crystal, which link the dimers into a column along the *b* axis (Fig. 3 ▸)

## Database survey   

A search of the CSD (Version 5.36; Groom & Allen 2014 ▸) gave three structures (VAHYAE: Dunstan *et al.*, 1998 ▸; FIVQAD Liu *et al.*, 2005 ▸; CUHHET: How *et al.*, 2009 ▸) closely related to the title compound. *S*-benzyl-β-*N*-(2-hy­droxy­phenyl­ethyl­idene)di­thio­carbazate was prepared by Pramanik *et al.* (2007 ▸) and its crystal structure was reported by Biswal *et al.* (2015 ▸).

## Synthesis and crystallization   

The ligand precursor, *S*-benzyl di­thio­carbazate (SBDTC), was prepared according to the literature method (Ali & Tarafder, 1977 ▸). The title compound was prepared as follows: to the ligand precursor, SBDTC (0.99 g, 5 mmol) dissolved in ethanol (40 ml) was added 2-hy­droxy aceto­phenone (0.68 g, 5 mmol) and the aliquot was heated under reflux for an 1h. The resultant yellow solution was cooled to room temperature. The light-yellow precipitate which formed was filtered off, washed with hot ethanol and dried under vacuum over anhydrous CaCl_2_ (yield: 1.23 g, 73.65%). The prepared compound (0.17 g) was dissolved in aceto­nitrile (20 ml) on warming and mixed with ethanol (10 ml). Light-yellow platelet single crystals of the title compound (m.p. 386–387 K) suitable for X-ray study were obtained after 17 days along with colorless needle-shaped crystalline solids (m.p. 413–418 K).

## Refinement   

Crystal data, data collection and structure refinement details are summarized in Table 2 ▸. All H atoms were positioned geometrically (C—H = 0.95–0.98 Å and O—H = 0.84 Å) and treated as riding with *U*_iso_(H) = 1.2*U*_eq_(C,O).

## Supplementary Material

Crystal structure: contains datablock(s) General, I. DOI: 10.1107/S2056989016002371/is5440sup1.cif

Structure factors: contains datablock(s) I. DOI: 10.1107/S2056989016002371/is5440Isup2.hkl

Supporting information file. DOI: 10.1107/S2056989016002371/is5440Isup3.cml

CCDC reference: 1452193

Additional supporting information:  crystallographic information; 3D view; checkCIF report

## Figures and Tables

**Table 1:** Hydrogen-bond geometry (Å, °) *Cg*1 is the centroid of the C1–C6 ring.

*D*—H⋯*A*	*D*—H	H⋯*A*	*D*⋯*A*	*D*—H⋯*A*
O1—H1⋯N1	0.84	1.84	2.5725 (15)	145
O2—H2⋯N4	0.84	1.84	2.576 (3)	146
O1—H1⋯S2	0.84	2.73	3.4112 (12)	139
O2—H2⋯S3	0.84	2.78	3.4792 (14)	141
C18—H18⋯O1^i^	0.99	2.52	3.4750 (19)	161
C18—H17⋯*Cg*1^ii^	0.99	2.54	3.5123 (17)	165

Symmetry codes: (i) 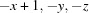; (ii) 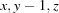.

**Table 2:** Experimental details

Crystal data	
Chemical formula	C_32_H_30_N_4_O_2_S_4_
*M*_r_	630.85
Crystal system, space group	Triclinic, *P* 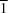
Temperature (K)	173
*a*, *b*, *c* (Å)	10.5556 (3), 11.0236 (3), 15.5261 (5)
α, β, γ (°)	75.9922 (8), 71.9673 (7), 65.5889 (7)
*V* (Å^3^)	1550.67 (8)
*Z*	2
Radiation type	Mo *K*α
μ (mm^−1^)	0.34
Crystal size (mm)	0.41 × 0.33 × 0.20

Data collection	
Diffractometer	Rigaku R-AXIS RAPID
Absorption correction	Multi-scan (*ABSCOR*; Higashi, 1995 ▸)
*T*_min_, *T*_max_	0.842, 0.935
No. of measured, independent and observed [*F* ^2^ > 2.0σ(*F* ^2^)] reflections	15605, 7075, 6443
*R*_int_	0.024
(sin θ/λ)_max_ (Å^−1^)	0.649

Refinement	
*R*[*F* ^2^ > 2σ(*F* ^2^)], *wR*(*F* ^2^), *S*	0.034, 0.093, 1.09
No. of reflections	7075
No. of parameters	383
H-atom treatment	H-atom parameters constrained
Δρ_max_, Δρ_min_ (e Å^−3^)	0.39, −0.35

Computer programs: *RAPID-AUTO* (Rigaku, 2001 ▸), *SIR92* (Altomare *et al.*, 1993 ▸), *SHELXL97* (Sheldrick, 2008 ▸) and *CrystalStructure* (Rigaku, 2010 ▸).

## supplementary crystallographic information

### Crystal data

**Table:**

C_32_H_30_N_4_O_2_S_4_	*Z* = 2
*M**_r_* = 630.85	*F*(000) = 660.00
Triclinic, *P*1	*D*_x_ = 1.351 Mg m^−^^3^
Hall symbol: -P 1	Mo *K*α radiation, λ = 0.71075 Å
*a* = 10.5556 (3) Å	Cell parameters from 14309 reflections
*b* = 11.0236 (3) Å	θ = 3.0–27.5°
*c* = 15.5261 (5) Å	µ = 0.34 mm^−^^1^
α = 75.9922 (8)°	*T* = 173 K
β = 71.9673 (7)°	Chunk, yellow
γ = 65.5889 (7)°	0.41 × 0.33 × 0.20 mm
*V* = 1550.67 (8) Å^3^	

### Data collection

**Table:**

Rigaku R-AXIS RAPID diffractometer	6443 reflections with *F*^2^ > 2.0σ(*F*^2^)
Detector resolution: 10.000 pixels mm^-1^	*R*_int_ = 0.024
ω scans	θ_max_ = 27.5°
Absorption correction: multi-scan (*ABSCOR*; Higashi, 1995)	*h* = −13→11
*T*_min_ = 0.842, *T*_max_ = 0.935	*k* = −14→14
15605 measured reflections	*l* = −20→20
7075 independent reflections	

### Refinement

**Table:**

Refinement on *F*^2^	Secondary atom site location: difference Fourier map
*R*[*F*^2^ > 2σ(*F*^2^)] = 0.034	Hydrogen site location: inferred from neighbouring sites
*wR*(*F*^2^) = 0.093	H-atom parameters constrained
*S* = 1.09	*w* = 1/[σ^2^(*F*_o_^2^) + (0.052*P*)^2^ + 0.2991*P*] where *P* = (*F*_o_^2^ + 2*F*_c_^2^)/3
7075 reflections	(Δ/σ)_max_ < 0.001
383 parameters	Δρ_max_ = 0.39 e Å^−^^3^
0 restraints	Δρ_min_ = −0.35 e Å^−^^3^
Primary atom site location: structure-invariant direct methods	

### Special details

**Table:**

Geometry. All s.u.'s (except the s.u. in the dihedral angle between two l.s. planes) are stimated using the full convariance matrix. The cell s.u.'s are taken into account individually in the estimation of s.u.'s in distances, angles and torsion angles; corrections between s.u.'s in cell parameters are only used when they are defined by crystal symmetry. An approximate (isotropic) treatment of cell s.u.'s is used for estimating s.u.'s involving l.s. planes.
Refinement. Refinement was performed using all reflections. The weighted *R*-factor (*wR*) and goodness of fit (*S*) are based on *F*^2^. *R*-factor (gt) are based on *F*. The threshold expression of *F*^2^ > 2.0 σ(*F*^2^) is used only for calculating *R*-factor (gt).

### Fractional atomic coordinates and isotropic or equivalent isotropic displacement parameters (Å^2^)

**Table:**

	*x*	*y*	*z*	*U*_iso_*/*U*_eq_	
S1	0.09853 (4)	0.18474 (3)	0.26158 (2)	0.04004 (9)	
S2	0.33374 (3)	0.22999 (3)	0.10197 (2)	0.03546 (9)	
S3	0.43320 (3)	0.06584 (3)	0.18428 (2)	0.03183 (8)	
S4	0.33603 (3)	−0.04421 (3)	0.06498 (2)	0.03407 (9)	
O1	0.34089 (12)	0.48991 (10)	−0.06903 (8)	0.0512 (3)	
O2	0.65619 (13)	−0.09530 (10)	0.33489 (8)	0.0490 (3)	
N1	0.12722 (11)	0.47399 (10)	0.06318 (7)	0.0301 (2)	
N2	0.06147 (11)	0.40274 (11)	0.13886 (7)	0.0328 (3)	
N3	0.48465 (11)	−0.19327 (10)	0.18757 (7)	0.0314 (2)	
N4	0.55167 (11)	−0.19781 (10)	0.25420 (7)	0.0296 (2)	
C1	0.26385 (16)	0.62281 (13)	−0.08014 (9)	0.0363 (3)	
C2	0.32953 (18)	0.70237 (15)	−0.14798 (10)	0.0444 (4)	
C3	0.2587 (2)	0.83934 (16)	−0.16257 (11)	0.0504 (4)	
C4	0.1229 (2)	0.89887 (16)	−0.11067 (14)	0.0588 (5)	
C5	0.05661 (18)	0.82114 (14)	−0.04409 (12)	0.0492 (4)	
C6	0.12461 (14)	0.68087 (12)	−0.02668 (9)	0.0332 (3)	
C7	0.05349 (13)	0.60139 (12)	0.04772 (9)	0.0317 (3)	
C8	−0.09410 (15)	0.66931 (15)	0.10333 (11)	0.0444 (4)	
C9	0.15035 (13)	0.28791 (12)	0.16418 (8)	0.0309 (3)	
C10	−0.08614 (15)	0.29472 (15)	0.30185 (10)	0.0452 (4)	
C11	−0.14947 (14)	0.22557 (13)	0.39113 (9)	0.0363 (3)	
C12	−0.25928 (15)	0.18530 (16)	0.39580 (10)	0.0431 (3)	
C13	−0.32091 (17)	0.12327 (17)	0.47688 (12)	0.0530 (4)	
C14	−0.27240 (19)	0.09964 (17)	0.55326 (11)	0.0567 (5)	
C15	−0.1638 (3)	0.1396 (2)	0.55012 (11)	0.0635 (5)	
C16	−0.10211 (19)	0.20308 (18)	0.46934 (11)	0.0522 (4)	
C17	0.42626 (12)	−0.07400 (12)	0.14963 (8)	0.0278 (3)	
C18	0.34252 (15)	−0.21244 (14)	0.06891 (9)	0.0367 (3)	
C19	0.24950 (14)	−0.26450 (14)	0.15237 (8)	0.0335 (3)	
C20	0.27934 (16)	−0.40188 (15)	0.16988 (10)	0.0409 (3)	
C21	0.19396 (18)	−0.45535 (16)	0.24294 (10)	0.0466 (4)	
C22	0.07643 (17)	−0.37179 (17)	0.29866 (10)	0.0465 (4)	
C23	0.04675 (16)	−0.23501 (17)	0.28200 (11)	0.0469 (4)	
C24	0.13335 (15)	−0.18127 (15)	0.20996 (10)	0.0399 (3)	
C25	0.58044 (12)	−0.30944 (12)	0.30954 (8)	0.0286 (3)	
C26	0.54341 (18)	−0.42358 (14)	0.30248 (11)	0.0450 (4)	
C27	0.64827 (12)	−0.31886 (12)	0.38200 (8)	0.0292 (3)	
C28	0.68143 (14)	−0.21190 (13)	0.39196 (9)	0.0333 (3)	
C29	0.74129 (15)	−0.22298 (15)	0.46335 (10)	0.0412 (3)	
C30	0.77032 (17)	−0.33862 (17)	0.52459 (10)	0.0459 (4)	
C31	0.73998 (18)	−0.44566 (17)	0.51615 (11)	0.0493 (4)	
C32	0.67943 (16)	−0.43490 (14)	0.44601 (10)	0.0398 (3)	
H1	0.2919	0.4508	−0.0280	0.0614*	
H2	0.6198	−0.0986	0.2949	0.0587*	
H3	0.4231	0.6618	−0.1840	0.0533*	
H4	0.3036	0.8930	−0.2086	0.0604*	
H5	0.0748	0.9935	−0.1206	0.0705*	
H6	−0.0373	0.8636	−0.0092	0.0591*	
H7	−0.1566	0.7271	0.0624	0.0533*	
H8	−0.0898	0.7239	0.1429	0.0533*	
H9	−0.1324	0.6012	0.1411	0.0533*	
H10	−0.0891	0.3817	0.3111	0.0542*	
H11	−0.1417	0.3122	0.2562	0.0542*	
H12	−0.2931	0.2003	0.3427	0.0517*	
H13	−0.3972	0.0972	0.4792	0.0635*	
H14	−0.3136	0.0557	0.6085	0.0680*	
H15	−0.1304	0.1237	0.6036	0.0762*	
H16	−0.0274	0.2310	0.4678	0.0626*	
H17	0.3147	−0.2148	0.0142	0.0440*	
H18	0.4430	−0.2752	0.0644	0.0440*	
H19	0.3590	−0.4600	0.1315	0.0491*	
H20	0.2164	−0.5497	0.2547	0.0560*	
H21	0.0168	−0.4080	0.3479	0.0558*	
H22	−0.0337	−0.1771	0.3202	0.0563*	
H23	0.1131	−0.0873	0.2000	0.0479*	
H24	0.4454	−0.4115	0.3380	0.0539*	
H25	0.6101	−0.5085	0.3265	0.0539*	
H26	0.5506	−0.4253	0.2383	0.0539*	
H27	0.7622	−0.1501	0.4697	0.0495*	
H28	0.8113	−0.3452	0.5728	0.0551*	
H29	0.7606	−0.5257	0.5582	0.0592*	
H30	0.6583	−0.5083	0.4410	0.0478*	

### Atomic displacement parameters (Å^2^)

**Table:**

	*U*^11^	*U*^22^	*U*^33^	*U*^12^	*U*^13^	*U*^23^
S1	0.03573 (18)	0.03325 (17)	0.03854 (18)	−0.01161 (13)	−0.00120 (13)	0.00559 (13)
S2	0.02997 (16)	0.02973 (16)	0.03605 (17)	−0.00918 (12)	−0.00505 (12)	0.00752 (12)
S3	0.03227 (16)	0.02733 (15)	0.03573 (16)	−0.01093 (12)	−0.01294 (12)	0.00250 (11)
S4	0.03448 (17)	0.03974 (17)	0.02849 (15)	−0.01494 (13)	−0.01321 (12)	0.00453 (12)
O1	0.0525 (7)	0.0291 (5)	0.0504 (6)	−0.0123 (5)	0.0081 (5)	0.0011 (5)
O2	0.0697 (8)	0.0361 (5)	0.0575 (7)	−0.0282 (5)	−0.0396 (6)	0.0129 (5)
N1	0.0316 (5)	0.0271 (5)	0.0319 (5)	−0.0112 (4)	−0.0111 (4)	0.0013 (4)
N2	0.0318 (6)	0.0308 (5)	0.0336 (6)	−0.0120 (5)	−0.0077 (5)	0.0005 (4)
N3	0.0327 (6)	0.0298 (5)	0.0317 (5)	−0.0097 (4)	−0.0127 (4)	−0.0005 (4)
N4	0.0297 (5)	0.0275 (5)	0.0304 (5)	−0.0085 (4)	−0.0112 (4)	−0.0005 (4)
C1	0.0509 (8)	0.0292 (6)	0.0328 (6)	−0.0177 (6)	−0.0165 (6)	0.0027 (5)
C2	0.0642 (10)	0.0437 (8)	0.0342 (7)	−0.0305 (7)	−0.0161 (7)	0.0045 (6)
C3	0.0827 (12)	0.0453 (8)	0.0449 (8)	−0.0409 (9)	−0.0389 (8)	0.0184 (7)
C4	0.0758 (12)	0.0299 (7)	0.0794 (12)	−0.0194 (8)	−0.0495 (11)	0.0180 (8)
C5	0.0497 (9)	0.0294 (7)	0.0694 (11)	−0.0078 (6)	−0.0339 (8)	0.0059 (7)
C6	0.0416 (7)	0.0270 (6)	0.0373 (7)	−0.0125 (5)	−0.0242 (6)	0.0035 (5)
C7	0.0326 (6)	0.0291 (6)	0.0365 (6)	−0.0085 (5)	−0.0180 (5)	−0.0023 (5)
C8	0.0361 (7)	0.0366 (7)	0.0529 (9)	−0.0028 (6)	−0.0143 (6)	−0.0065 (6)
C9	0.0308 (6)	0.0300 (6)	0.0314 (6)	−0.0132 (5)	−0.0062 (5)	−0.0009 (5)
C10	0.0330 (7)	0.0435 (8)	0.0435 (8)	−0.0098 (6)	−0.0025 (6)	0.0054 (6)
C11	0.0338 (7)	0.0347 (7)	0.0335 (7)	−0.0099 (6)	−0.0024 (5)	−0.0040 (5)
C12	0.0388 (8)	0.0491 (8)	0.0399 (7)	−0.0165 (7)	−0.0046 (6)	−0.0088 (6)
C13	0.0425 (8)	0.0479 (9)	0.0595 (10)	−0.0204 (7)	0.0064 (7)	−0.0085 (8)
C14	0.0595 (10)	0.0454 (9)	0.0407 (8)	−0.0146 (8)	0.0136 (7)	−0.0042 (7)
C15	0.0872 (14)	0.0642 (11)	0.0331 (8)	−0.0215 (10)	−0.0174 (8)	−0.0033 (8)
C16	0.0597 (10)	0.0561 (10)	0.0486 (9)	−0.0271 (8)	−0.0195 (8)	−0.0013 (7)
C17	0.0252 (6)	0.0313 (6)	0.0255 (5)	−0.0109 (5)	−0.0057 (5)	−0.0006 (5)
C18	0.0411 (7)	0.0453 (8)	0.0290 (6)	−0.0198 (6)	−0.0093 (5)	−0.0061 (6)
C19	0.0356 (7)	0.0425 (7)	0.0296 (6)	−0.0183 (6)	−0.0147 (5)	−0.0012 (5)
C20	0.0491 (8)	0.0422 (8)	0.0361 (7)	−0.0167 (7)	−0.0166 (6)	−0.0047 (6)
C21	0.0651 (10)	0.0441 (8)	0.0427 (8)	−0.0284 (8)	−0.0250 (7)	0.0045 (6)
C22	0.0517 (9)	0.0599 (10)	0.0384 (7)	−0.0335 (8)	−0.0164 (7)	0.0063 (7)
C23	0.0386 (8)	0.0572 (9)	0.0447 (8)	−0.0220 (7)	−0.0045 (6)	−0.0052 (7)
C24	0.0372 (7)	0.0418 (8)	0.0418 (7)	−0.0171 (6)	−0.0098 (6)	−0.0022 (6)
C25	0.0258 (6)	0.0252 (6)	0.0307 (6)	−0.0063 (5)	−0.0072 (5)	−0.0014 (5)
C26	0.0611 (9)	0.0325 (7)	0.0517 (9)	−0.0217 (7)	−0.0298 (8)	0.0059 (6)
C27	0.0256 (6)	0.0287 (6)	0.0295 (6)	−0.0074 (5)	−0.0073 (5)	−0.0008 (5)
C28	0.0323 (6)	0.0310 (6)	0.0360 (7)	−0.0112 (5)	−0.0117 (5)	0.0006 (5)
C29	0.0438 (8)	0.0448 (8)	0.0416 (7)	−0.0195 (7)	−0.0160 (6)	−0.0033 (6)
C30	0.0499 (9)	0.0573 (9)	0.0357 (7)	−0.0226 (7)	−0.0200 (6)	0.0029 (7)
C31	0.0614 (10)	0.0489 (9)	0.0411 (8)	−0.0243 (8)	−0.0264 (7)	0.0142 (7)
C32	0.0475 (8)	0.0344 (7)	0.0398 (7)	−0.0175 (6)	−0.0182 (6)	0.0059 (6)

### Geometric parameters (Å, º)

**Table:**

S1—C9	1.7461 (12)	C25—C27	1.473 (2)
S1—C10	1.8220 (13)	C27—C28	1.414 (3)
S2—S3	2.0373 (4)	C27—C32	1.4041 (18)
S2—C9	1.7909 (12)	C28—C29	1.395 (3)
S3—C17	1.7862 (16)	C29—C30	1.376 (2)
S4—C17	1.7464 (15)	C30—C31	1.388 (4)
S4—C18	1.8139 (18)	C31—C32	1.384 (3)
O1—C1	1.3480 (16)	O1—H1	0.840
O2—C28	1.3491 (16)	O2—H2	0.840
N1—N2	1.4065 (15)	C2—H3	0.950
N1—C7	1.2980 (15)	C3—H4	0.950
N2—C9	1.2818 (15)	C4—H5	0.950
N3—N4	1.4043 (19)	C5—H6	0.950
N3—C17	1.2789 (15)	C8—H7	0.980
N4—C25	1.2983 (15)	C8—H8	0.980
C1—C2	1.401 (3)	C8—H9	0.980
C1—C6	1.4082 (18)	C10—H10	0.990
C2—C3	1.377 (2)	C10—H11	0.990
C3—C4	1.377 (3)	C12—H12	0.950
C4—C5	1.383 (3)	C13—H13	0.950
C5—C6	1.4070 (18)	C14—H14	0.950
C6—C7	1.4773 (19)	C15—H15	0.950
C7—C8	1.4997 (17)	C16—H16	0.950
C10—C11	1.5117 (19)	C18—H17	0.990
C11—C12	1.380 (3)	C18—H18	0.990
C11—C16	1.383 (3)	C20—H19	0.950
C12—C13	1.385 (3)	C21—H20	0.950
C13—C14	1.361 (3)	C22—H21	0.950
C14—C15	1.373 (4)	C23—H22	0.950
C15—C16	1.390 (3)	C24—H23	0.950
C18—C19	1.5135 (19)	C26—H24	0.980
C19—C20	1.388 (3)	C26—H25	0.980
C19—C24	1.3904 (18)	C26—H26	0.980
C20—C21	1.389 (3)	C29—H27	0.950
C21—C22	1.383 (2)	C30—H28	0.950
C22—C23	1.382 (3)	C31—H29	0.950
C23—C24	1.390 (3)	C32—H30	0.950
C25—C26	1.499 (3)		
			
C9—S1—C10	99.56 (6)	C28—O2—H2	109.466
S3—S2—C9	104.38 (4)	C1—C2—H3	119.943
S2—S3—C17	105.39 (5)	C3—C2—H3	119.937
C17—S4—C18	99.70 (7)	C2—C3—H4	119.877
N2—N1—C7	115.35 (9)	C4—C3—H4	119.879
N1—N2—C9	112.35 (10)	C3—C4—H5	119.948
N4—N3—C17	113.08 (13)	C5—C4—H5	119.947
N3—N4—C25	114.85 (13)	C4—C5—H6	119.129
O1—C1—C2	116.61 (12)	C6—C5—H6	119.136
O1—C1—C6	122.57 (13)	C7—C8—H7	109.476
C2—C1—C6	120.82 (12)	C7—C8—H8	109.462
C1—C2—C3	120.12 (14)	C7—C8—H9	109.472
C2—C3—C4	120.24 (16)	H7—C8—H8	109.476
C3—C4—C5	120.10 (14)	H7—C8—H9	109.473
C4—C5—C6	121.73 (14)	H8—C8—H9	109.468
C1—C6—C5	116.97 (13)	S1—C10—H10	110.101
C1—C6—C7	122.56 (11)	S1—C10—H11	110.109
C5—C6—C7	120.41 (11)	C11—C10—H10	110.096
N1—C7—C6	116.53 (10)	C11—C10—H11	110.108
N1—C7—C8	123.33 (12)	H10—C10—H11	108.444
C6—C7—C8	120.10 (11)	C11—C12—H12	119.542
S1—C9—S2	117.77 (6)	C13—C12—H12	119.538
S1—C9—N2	121.43 (9)	C12—C13—H13	119.911
S2—C9—N2	120.78 (9)	C14—C13—H13	119.899
S1—C10—C11	107.98 (9)	C13—C14—H14	120.123
C10—C11—C12	119.49 (15)	C15—C14—H14	120.120
C10—C11—C16	121.90 (17)	C14—C15—H15	119.748
C12—C11—C16	118.60 (14)	C16—C15—H15	119.742
C11—C12—C13	120.92 (17)	C11—C16—H16	119.988
C12—C13—C14	120.2 (2)	C15—C16—H16	120.002
C13—C14—C15	119.76 (16)	S4—C18—H17	108.133
C14—C15—C16	120.5 (2)	S4—C18—H18	108.131
C11—C16—C15	120.0 (3)	C19—C18—H17	108.132
S3—C17—S4	118.82 (7)	C19—C18—H18	108.138
S3—C17—N3	120.15 (12)	H17—C18—H18	107.308
S4—C17—N3	121.03 (13)	C19—C20—H19	119.639
S4—C18—C19	116.64 (10)	C21—C20—H19	119.640
C18—C19—C20	117.90 (11)	C20—C21—H20	119.866
C18—C19—C24	123.27 (13)	C22—C21—H20	119.868
C20—C19—C24	118.79 (13)	C21—C22—H21	120.357
C19—C20—C21	120.72 (13)	C23—C22—H21	120.354
C20—C21—C22	120.27 (16)	C22—C23—H22	119.677
C21—C22—C23	119.29 (15)	C24—C23—H22	119.671
C22—C23—C24	120.65 (13)	C19—C24—H23	119.868
C19—C24—C23	120.25 (15)	C23—C24—H23	119.878
N4—C25—C26	122.05 (14)	C25—C26—H24	109.480
N4—C25—C27	117.20 (14)	C25—C26—H25	109.474
C26—C25—C27	120.73 (11)	C25—C26—H26	109.469
C25—C27—C28	122.18 (11)	H24—C26—H25	109.472
C25—C27—C32	120.68 (15)	H24—C26—H26	109.464
C28—C27—C32	117.12 (15)	H25—C26—H26	109.468
O2—C28—C27	122.60 (15)	C28—C29—H27	119.731
O2—C28—C29	116.91 (16)	C30—C29—H27	119.725
C27—C28—C29	120.48 (12)	C29—C30—H28	119.862
C28—C29—C30	120.54 (18)	C31—C30—H28	119.853
C29—C30—C31	120.28 (18)	C30—C31—H29	120.257
C30—C31—C32	119.47 (15)	C32—C31—H29	120.269
C27—C32—C31	122.09 (17)	C27—C32—H30	118.957
C1—O1—H1	109.472	C31—C32—H30	118.953
			
C9—S1—C10—C11	−176.67 (11)	S1—C10—C11—C12	−114.03 (11)
C10—S1—C9—S2	175.25 (11)	S1—C10—C11—C16	67.04 (15)
C10—S1—C9—N2	−3.17 (15)	C10—C11—C12—C13	−179.14 (11)
S3—S2—C9—S1	−9.83 (11)	C10—C11—C16—C15	179.74 (11)
S3—S2—C9—N2	168.60 (11)	C12—C11—C16—C15	0.8 (2)
C9—S2—S3—C17	91.54 (6)	C16—C11—C12—C13	−0.2 (2)
S2—S3—C17—S4	−3.18 (7)	C11—C12—C13—C14	−0.8 (3)
S2—S3—C17—N3	177.93 (7)	C12—C13—C14—C15	1.1 (3)
C17—S4—C18—C19	70.94 (11)	C13—C14—C15—C16	−0.5 (3)
C18—S4—C17—S3	−172.82 (7)	C14—C15—C16—C11	−0.5 (3)
C18—S4—C17—N3	6.06 (10)	S4—C18—C19—C20	−162.98 (10)
N2—N1—C7—C6	175.20 (12)	S4—C18—C19—C24	19.4 (2)
N2—N1—C7—C8	−2.3 (3)	C18—C19—C20—C21	−177.02 (14)
C7—N1—N2—C9	−164.04 (13)	C18—C19—C24—C23	175.75 (14)
N1—N2—C9—S1	175.36 (12)	C20—C19—C24—C23	−1.9 (3)
N1—N2—C9—S2	−3.0 (2)	C24—C19—C20—C21	0.7 (3)
N4—N3—C17—S3	−0.88 (13)	C19—C20—C21—C22	0.9 (3)
N4—N3—C17—S4	−179.74 (8)	C20—C21—C22—C23	−1.4 (3)
C17—N3—N4—C25	163.60 (9)	C21—C22—C23—C24	0.2 (3)
N3—N4—C25—C26	−0.41 (14)	C22—C23—C24—C19	1.4 (3)
N3—N4—C25—C27	−178.84 (8)	N4—C25—C27—C28	0.01 (14)
O1—C1—C2—C3	−178.99 (16)	N4—C25—C27—C32	178.36 (9)
O1—C1—C6—C5	178.96 (15)	C26—C25—C27—C28	−178.44 (10)
O1—C1—C6—C7	1.9 (3)	C26—C25—C27—C32	−0.09 (15)
C2—C1—C6—C5	−0.7 (3)	C25—C27—C28—O2	−1.44 (16)
C2—C1—C6—C7	−177.74 (16)	C25—C27—C28—C29	177.84 (9)
C6—C1—C2—C3	0.7 (3)	C25—C27—C32—C31	−178.45 (9)
C1—C2—C3—C4	−0.1 (3)	C28—C27—C32—C31	−0.02 (17)
C2—C3—C4—C5	−0.5 (4)	C32—C27—C28—O2	−179.85 (10)
C3—C4—C5—C6	0.5 (4)	C32—C27—C28—C29	−0.57 (16)
C4—C5—C6—C1	0.1 (3)	O2—C28—C29—C30	179.98 (10)
C4—C5—C6—C7	177.23 (19)	C27—C28—C29—C30	0.67 (17)
C1—C6—C7—N1	1.4 (3)	C28—C29—C30—C31	−0.16 (19)
C1—C6—C7—C8	178.99 (15)	C29—C30—C31—C32	−0.4 (2)
C5—C6—C7—N1	−175.60 (16)	C30—C31—C32—C27	0.5 (2)
C5—C6—C7—C8	2.0 (3)		

### Hydrogen-bond geometry (Å, º)

*Cg*1 is the centroid of the C1–C6 ring.

**Table:**

*D*—H···*A*	*D*—H	H···*A*	*D*···*A*	*D*—H···*A*
O1—H1···N1	0.84	1.84	2.5725 (15)	145
O2—H2···N4	0.84	1.84	2.576 (3)	146
O1—H1···S2	0.84	2.73	3.4112 (12)	139
O2—H2···S3	0.84	2.78	3.4792 (14)	141
C18—H18···O1^i^	0.99	2.52	3.4750 (19)	161
C18—H17···*Cg*1^ii^	0.99	2.54	3.5123 (17)	165

Symmetry codes: (i) −*x*+1, −*y*, −*z*; (ii) *x*, *y*−1, *z*.
